# Isokinetic Strength and Functional Ratio for Quadriceps and Hamstrings among Semi-Professional Athletes in UAE playing Football, Cricket, and Tennis- Biomechanical Implications

**DOI:** 10.12688/f1000research.170911.1

**Published:** 2025-10-24

**Authors:** Yogi Bhatt, Animesh Hazari, Shashikumar Channmgere Govindappa, Praveen Kumar Kandakurti

**Affiliations:** 1 College of Health Sciences, Gulf Medical University, Ajman, Ajman, United Arab Emirates; 2 College of Applied Medical Sciences, University of Hail, Hail, Hail Province, Saudi Arabia

**Keywords:** Isokinetic strength, Hamstring, Quadriceps, Functional ratio, H/Q ratio

## Abstract

**Aim:**

This study aims to compare the isokinetic strength of the Hamstrings against the Quadriceps for Football, Cricket, and Tennis among semi-professional athletes.

**Methodology:**

An observational, cross-sectional study was conducted with the inclusion of participants between 18-40 years, both males and females having an active athlete profile playing Football, Cricket, and Tennis as semi-professional (competing in inter-collegiate, national-level tournaments in the UAE). The minimum duration for each sport was one hour per day and three to five times per week. A total of 66 participants were enrolled, with 22 participants in each group. The ISOMOVE isokinetic device was used to assess the strength of dynamically contracting Quadriceps concentrically against eccentric contraction of the Hamstring muscles to determine the functional ratio (H/Q ratio).

**Results:**

Age-wise distribution of players showed predominant male participants with 77.3% of players in Football, 68.2% in Cricket, and 59.1% in Tennis. The comparison for the mean peaks of isokinetic strength for functional ratio suggested a statistically significant difference between the three sports (Football p<0.001, Cricket p=0.006, Tennis p =0.003). The findings suggested that the highest H/Q ratio was found in Football players with a mean value of 0.51±0.07, followed by Cricket and Tennis with a mean of 0.48±0.08 and 0.42±0.05, respectively (p<0.05).

**Conclusion:**

The isokinetic strength testing for H/Q ratio was found to be higher in Football players, followed by Cricket and Tennis players for semi-professional athletes. The study concluded that the functional H/Q ratio varies between three similar sports as the demand on the target muscle would specifically vary. Thus, semi-professional players who play multiple games should consider enhancing FR for the Hamstring to the Quadriceps, besides the strength and training specific to sports, to avoid the risk of injuries.

## Introduction

Lower limb muscular strength is one of the important factors for performance and injury prevention in most outdoor sports. Monitoring the strength of the muscle could be essential for assessing and anticipating its functional capacity. ^1^ Enhancing lower limb muscle strength features a structured training regimen meant to improve the athletes’ tactical and technical abilities, which could enhance physical fitness. ^2^ Isokinetic strength evaluation (ISE) is amongst the most widely employed techniques for assessing lower limb muscle strength in all athletes. ^3^ The functional capacity, joint stability, muscular activity during velocity-dependent movements, and muscle coordination within each muscle group are all evaluated using the knee antagonist-to-agonist ratio. ^4^ Therefore, the Hamstring-to-Quadriceps relationship (H/Q ratio) relates the maximal torque of the Hamstring muscle to the Quadriceps muscle, serving a significant role in outdoor sports. ^4^ A study conducted on professional soccer players concluded that determining the isokinetic strength during preseason could be important in predicting strength imbalance as a risk factor for Hamstring injuries. ^5^ Because most of the movements involved in sports demand simultaneous concentric knee extension and eccentric knee flexion, eccentric Hamstring strength stated relative to concentric Quadricep strength is referred to as the “functional ratio” (FR). ^6^ The development of the functional Hamstring to Quadriceps H: Q ratio, as opposed to the more conventional H: Q ratio (CR), which compares the Hamstring concentric peak torque (PT) during leg flexion to the Quadriceps concentric PT during leg extension, was proposed as an improved measurement of the agonist: antagonist relationship. ^7^ The CR provides information on the muscular potential during dynamic stabilization of the knee joint whereas, ^7^ the FR is an indicator of the braking function of the Hamstrings during an extension of maximal Quadriceps strength as the co-activation of the Hamstrings is an important factor in maintaining stability of the knee joint as seen for various on field sports. ^6^ Regardless of the sport, most injuries sustained by athletes are Hamstring strains ^4, 8^ and can keep a person out of sports for an extended period. ^9^ According to the studies, an overall 1.2-4 lower limb injuries per 1000 hours of exposure for athletes, ^10, 11^ and disproportionate strength between the eccentric Hamstring and concentric Quadriceps muscles could be the main reason. ^12^ Studies report an annual increase of 4% in Hamstring injuries alone among all lower limb injuries in professional footballers since 2001. ^10^ Though the majority of studies support the role of the H/Q ratio in soft tissue injuries at the knee joint, a study recently concluded that isokinetic strength deficits in Hamstring and Quadriceps could be a weak risk factor for Hamstring strains. ^13^ Nevertheless, isokinetic strength testing in clinical practice is still popular among the athletic population.

The semi-professional players denote the physical and mental refreshing activities while often participating in college sports competitions. Sports like Football, Cricket, and Tennis have similar components in practice (lower limb strength) but may have different involvement in the functional H: Q ratio. Due to the nature of the activity, approximately 70% of soccer injuries occur in the lower limbs, with the knee being the most frequent site (54%). ^14^ Football players often present anterior cruciate ligament (ACL) injury following the unbalanced eccentric contraction of the Hamstrings and concentric contraction of the Quadriceps. ^15^ Similarly, at specific phases of bowling in cricket bowlers, altered co-contraction is commonly encountered, which can lead to injury. ^16^ In Tennis, the torque with disproportionate agonist and antagonist balance results in anterior translation of the tibia, thereby straining ligaments and other soft tissues at the knee joint. ^17^ Therefore, the isokinetic strength evaluation for functional H/Q ratio could enable the early determination of muscular imbalances leading to the risk of hamstring strain injuries (HSI). Comparing the ratio in these sports would help to differentiate and quantify the relative muscle strength and its impact on participation. Hence, this study aims to compare the isokinetic strength of the Hamstrings against the Quadriceps for Football, Cricket, and Tennis among semi-professional athletes under the following objectives:

1. To compare the differences in the ratio for eccentric Hamstring and concentric Quadriceps (H/Q ratio) among semi-professional athletes in UAE playing Football, Cricket, and Tennis.
2. To correlate the H/Q ratio between semi-professional athletes playing Football, Cricket, and Tennis.

## Methodology

### Study design and population

An observational, cross-sectional study was conducted with the following inclusion criteria:

• Active athletes playing Football, Cricket, and Tennis as semi-professional athletes.
• Participants within the age of 18 years to 40 years.
• Both males and females were included.
• The minimum duration for each sport was taken as one hour per day and three to five times per week continued for a minimum in the past 6 months.

Participants with an implant or recent surgeries of the lower limb were excluded. In addition, participants with an active pregnancy and acute musculoskeletal injuries hindering their regular sports sessions were also excluded.

A total of 226 semi-professional players were screened initially from the various universities within the United Arab Emirates, out of which 74 players (participants) were recruited based on the inclusion criteria. Further, a total of 12 participants were excluded due to the following reasons:

Acute lower limb injuries- n = 8

Active pregnancy- n = 3

Tibial fracture with surgical reduction- n = 1

### Sample size and sampling

A total of sixty-six participants enrolled, with 22 participants in each group under purposive sampling. The sample size was estimated assuming an 80% power and effect size of 0.5 at a 95% Confidence Interval using the formula for comparison of means (n = (Zα/2+Zβ)2 *2*σ2/d2). The H/Q ratio in percentage was taken as an independent variable set at 60°s ^−1^ for isokinetic strength testing among athletes. The critical values of 1.96 and 0.84 were used for Zα/2 and Zβ, respectively. The pooled standard deviation (σ) of 16% was calculated from the previous study and a minimal clinical difference of 10% was considered to yield a sample size of 22 in each group. ^18^ The total sample size aligned with a similar previous study. ^19^ A study has reported sample size calculation as a Rule of Thumb (ROT) that also supports our sample size in each group for statistical inferences. ^20^

### Study settings

The study was conducted at the Thumbay Physical Therapy and Rehabilitation Centre, Gulf Medical University, Ajman, United Arab Emirates.

### Ethical approval and procedure

The study protocol received approval from the Institutional Review Board (IRB-COHS-STD-57-APRIL-2023), Gulf Medical University, Ajman, UAE. All participants provided written informed consent, and the ethical standards for the project were met under the Declaration of Helsinki. The participants’ recruitment began on 13 ^th^ June 2023 and ended on 29 ^th^ Dec 2023. Demographics, including age, gender, and Body Mass Index (BMI), were taken for each participant. The ISOMOVE isokinetic system software 0.0.1 (ISO-MANSW-IT, Tecnobody, Italy) was utilized to determine the isokinetic strength of the Hamstring and Quadriceps muscles. It acts as an optimal system for isokinetic testing, essential to analyze the strength with high reliability and validity. ^21^ Before the initiation of data collection, all the participants were informed about the procedure and equipment. The shin pad was set at 5.1 cm (2 inches) superior to the medial malleolus. Participants were sitting and stabilized by safety belts across the chest, thighs, and hips to prevent anterior upper body movement. Measurements were taken at 90-degree knee flexion to 10-degree knee extension range. All participants were verbally instructed to keep their arms crossed over their chests and were motivated to exert maximum effort during the five-second contractions. Each test included 3 consecutive trials of 5 seconds with a 2-minute rest between the trials. ^22^ The torque was set at 60°.s ^−1^ for concentric Quadriceps and eccentric Hamstring isokinetic strength testing. ^23^

### Standardization

It was ensured that the entire methodology for data collection was standardized for all participants. The selection of participants was strictly followed to match their level of sports activity. For instance, participants from Football only included strikers and defenders (excluding goalkeepers due to fewer running tasks), and participants from Cricket included fast bowlers (excluding wicketkeepers, spinners, and batsmen). All tennis players demonstrate similar lower limb strength and endurance irrespective of the position of play, thus matching the levels of Football and Cricket.

Before the data collection, all participants were familiarized with the isokinetic strength testing device. ^23^ A pre-set protocol was followed as recommended by Brown. ^24^ The data was collected over the dominant lower limb only, and overall, 78% of participants were right-dominant, with 22% left-dominant. Before the measurement, all participants were given a 5-minute cycle ergometer warm-up and active dynamic stretching. ^25^ The recorded data was first transferred from the software to an Excel sheet. The peak score of the three readings was considered for statistical analysis after the data were normalized to body mass (Nm·kg ^−1^). ^26^

### Statistical analysis

The descriptive analysis was conducted to study the demographic characteristics. Following a normality test, one-way ANOVA was conducted to determine the mean difference between the groups. Pierson’s correlation test (r = -1 to 1) was carried out to determine the correlation for the H/Q ratio between the groups. The statistical significance was set at p < 0.05.

## Results

The groups were comparable with statistically non-significant demographic characteristics such as height and weight (p > 0.05). The mean Body Mass Index (BMI) was 21.23 ± 1.48, 22.08 ± 1.12, and 21.99 ± 0.95 for Cricket, Football, and Tennis, respectively ( Table 1).

**Table 1.** Demographic characteristics and professional findings.

Sports category	Age (Mean ± S.D)	Body Mass Index (BMI) (Mean ± S.D)	Duration of game excluding warm up and cool down (min/day)	Duration of game (days/week)	Years of Experience (Mean ± S.D)
Football (n = 22)	23.7 ± 12.9	21.23 ± 1.48	n = 7 (60 min/day) n = 15 (90 min/day)	n = 15 (3 days/week) n = 4 (4 days/week) n = 3 (5 days/week)	5.6 ± 2.3
Cricket (n = 22)	21.4 ± 12.3	22.08 ± 1.12	n = 3 (60 min/day) n = 17 (90 min/day) n = 2 (120 min/day)	n = 17 (3 days/week) n = 5 (5 days/week)	4.2 ± 1.8
Tennis (n = 22)	25 ± 13.5	21.99 ± 0.95	n = 19 (60 min/day) n = 2 (90 min/day) n = 1 (120 min/day)	n = 13 (3 days/week) n = 5 (4 days/week) n = 4 (5 days/week)	3.7 ± 1.4

**Figure 1.**
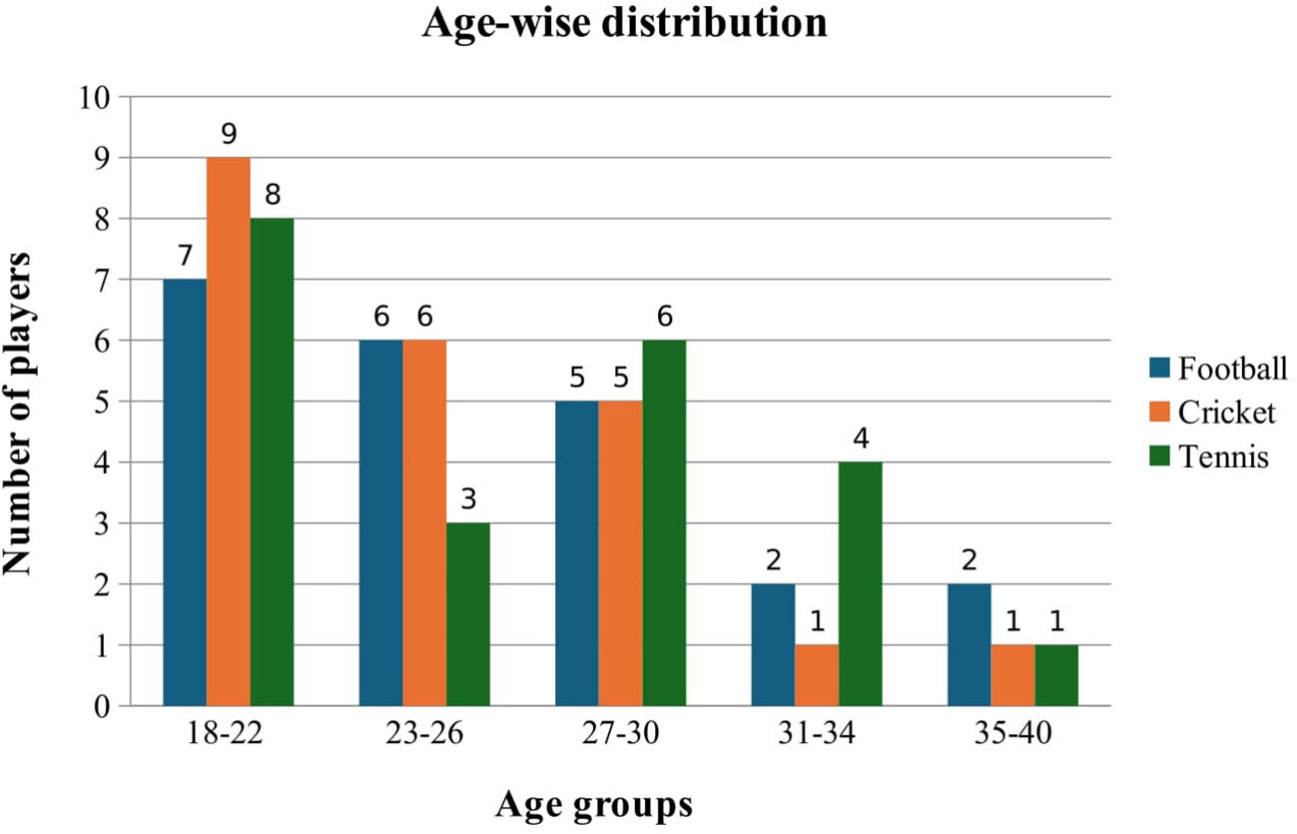
Age-wise distribution of participants.

Among Football players, the maximum number of participants was in the age group between 18-22 years, consisting of 31.8%, followed by 23-26 years (27.2%), 27-30 years (22.7%), 31-34 years, and 35-40 years (9.1%) respectively. Similarly, in Cricket, the maximum number of participants were in the age group between 18-22 years, consisting of 40.9%, followed by 27.2% under 23-26 years, 22.7% under 27-30 years, and 4.5% each under 31-34 years and 35-40 years age group. Furthermore, in Tennis also, the maximum number of participants were in the age group between 18-22 years (36.3%), followed by 27.2% under 27-30 years bracket, 13.6% under 23-26 years, 18.2% under 31-34 years, and 4.5% under 35-40 years ( Figure 1).

**Figure 2.**
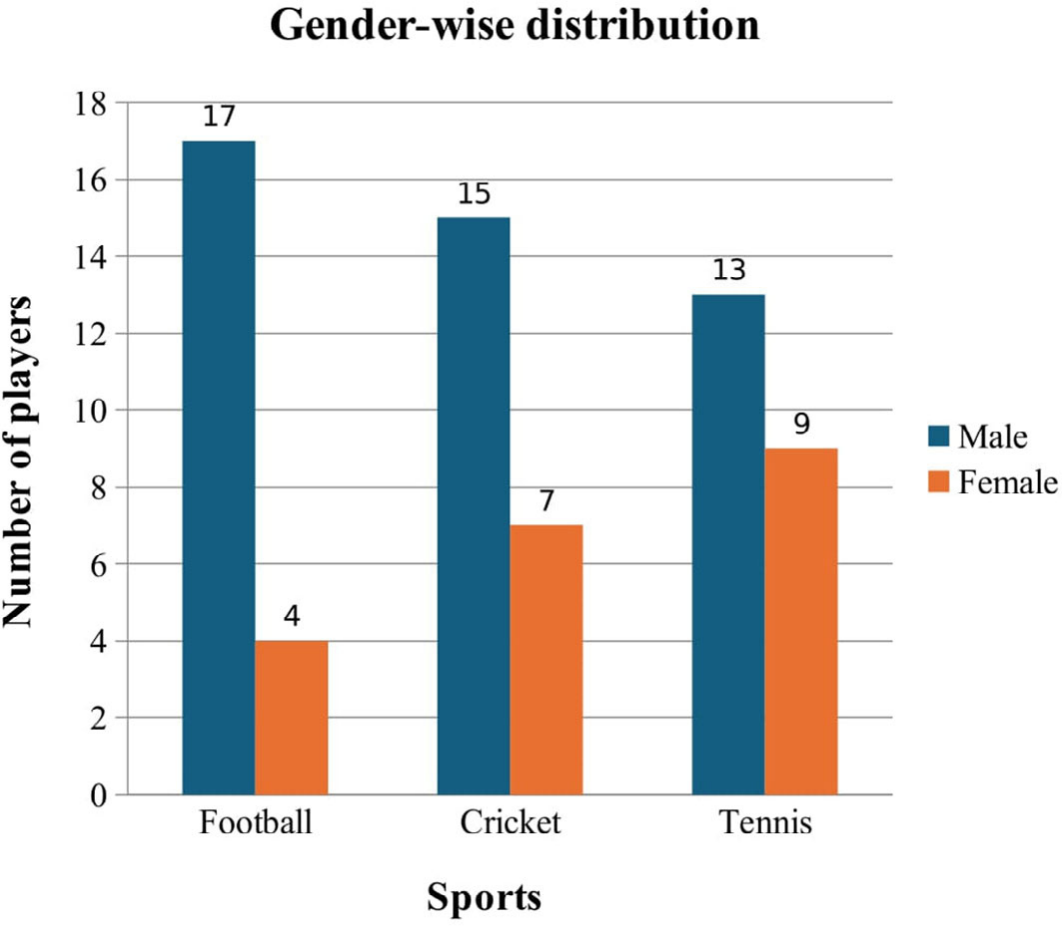
Gender-wise distribution of participants.

Figure 2 demonstrates that most players in Football were males, consisting of n = 17 (77.3%), whereas females contributed to n = 4 (18.2%). Similarly, in Cricket, the number of male and female players was n = 15 (68.2%), and n = 7 (31.8%) respectively. Additionally, in Tennis, the number of male players was n = 13 (59.1%), and n = 9 (31.8%) were females.

The mean of peaks of isokinetic strength of all three sports is given in Table 2, which suggests that a higher H/Q ratio was found in Football players with a mean value of 0.51 ± 0.07, followed by Cricket and Tennis with mean values of 0.48 ± 0.08 and 0.42 ± 0.05, respectively. When the one-way ANOVA test was applied, the p-value was found to be statistically significant (p < 0.05). Similar findings were found for females in three sports.

**Table 2.** Comparison of means for peak functional strength ratio (H/Q) between three sports following data normalization (Nm.Kg ^−1^).

Sport	H/Q ratio (Male) Mean ± SD	One-way ANOVA	H/Q ratio (Female) Mean ± SD	One-way ANOVA
**Football**	0.51 ± 0.07	<0.001	0.41 ± 0.12	0.031
**Cricket**	0.48 ± 0.08	0.006	0.33 ± 0.18	0.008
**Tennis**	0.42 ± 0.05	0.003	0.31 ± 0.09	0.005
**p-value **		p < 0.05, Significant		p < 0.05, Significant

The FR (H/Q ratio) for correlation between the groups was assessed by Pearson’s correlation test as given in Table 3. The results suggested that when the peak FR for Football was compared with the peak FR of Cricket, the r-value was 0.17 and the p-value was 0.44. Thus, it was found to be statistically insignificant. Similarly, when Cricket was correlated with Tennis, the r-value was 0.29, and the p-value was 0.19, suggesting insignificant statistical results. Also, when Football was compared with Tennis, findings were statistically insignificant (p = 0.08). Correlation analyses revealed that the athletes in all sports groups demonstrated a weak correlation for the H/Q ratio in isokinetic strength testing.

**Table 3.** Overall Functional Ratio (FR) correlation between the groups.

Sport	Pearson’s correlation (r-value)	p-value
H/Q of Football versus Cricket	0.17	0.44
H/Q Cricket versus Tennis	0.29	0.19
H/Q Football versus Tennis	0.38	0.08

## Discussion

Table 1 represents the demographic characteristics of all participants. It was found that the groups were comparable in age, height, and weight, and no statistically significant difference was found between the groups (p > 0.05). In the context of the duration of gameplay and professional experience, the groups showed similar levels of involvement in their respective games. The maximum duration of regular participation in the game on a single day was 120 minutes for Cricket and Tennis, whereas a maximum of 90 minutes was dedicated to Football ( Table 1). Moreover, participants in all three groups dedicated 3-5 days a week ( Table 1). The experience in the game ranged from 3 years to 7 years for each sport. Therefore, it could be said that their levels of physical activity were similar and comparable.

Figure 2 demonstrates the gender-wise distribution of players. Considering all three sports, 45 (68.2%) participants were males compared to females (n = 21, 31.8%), suggesting lower participation of females in these sports within the UAE, which is in consensus with a previous study conducted in Saudi Arabia. ^22^

The findings of our study suggest that when the means of peak isokinetic strength for functional (H/Q ratio) of all three sports were compared, FR was found to be maximum in Football participants (0.51 ± 0.07) followed by Cricket (0.48 ± 0.08) and Tennis (0.42 ± 0.05), which were statistically significant, p < 0.05 ( Table 2). A study conducted on 28 elite male soccer players reported an H/Q ratio of 64.7 ± 9.3 % for 5 reps at 60°s ^-1^, which was higher compared to our study and could be attributed to the difference in levels of the players (elite against semi-professional). ^27^ However, our findings agreed with the previous study, which concluded that a functional H/Q ratio of 52 ± 14% was observed among 18 semi-professional players for 3 reps at 60° s ^-1^. ^19^ Similar findings for females suggested that engaging in football led to a higher FR among female athletes compared to Cricket and Tennis. However, the gender analysis also suggested a lower FR for females compared to males involved in the same three sports ( Table 2), which was in line with a previous study. ^28^ The data on the H/Q ratio for Cricket and Tennis is scarce, although the rate of knee injuries is clinically significant. A study conducted in southern India ^16^ concluded that isokinetic strength among Cricket fast bowlers could be well assessed by eccentric to concentric H/Q ratio, as reported in our study. Although the study did not specify the mean H/Q ratio, a significant difference in isokinetic strength was seen for pre- and post-plyometric-based training, suggesting that the H/Q ratio is an important strength variable for Cricket fast bowlers. ^16^ Comparing Tennis as a court game similar to Basketball, a study reported an H/Q ratio of 0.52 (1.68 ± 0.24/3.18 ± 0.54 Nm.Kg ^−1^) at 60° s ^-1^. ^29^ The ratio was higher compared to our study for Tennis, and the reason could be attributed analysis of CR in the previous study to FR in our study. We assume that the ratio could be similar for FR, as both represent the court games category with similar demands of lower limb strength.

As muscle strength imbalance around the knee joint has been long considered a risk factor for hamstring strain injury (HSI) and is typically examined with the functional (H/Q) ratio, ^30^ the findings of this study suggested that all three groups could be at risk of injury since the peak force of Quadriceps was higher than Hamstring. In other words, a muscular force imbalance was observed for the eccentric contraction of the Hamstring to the concentric Quadriceps, resulting in a lower FR for all sports. However, the H/Q ratio was highest in the Football groups, suggesting a lower comparative risk of knee injuries. Studies report that a balanced strength of the Hamstring and Quadriceps makes it possible to identify the muscular performance profiles of Football players, making it important for both maximizing physical performance and avoiding injuries. ^31^ To prevent Hamstring and/or knee-related problems, previous studies on adults suggested that the typical H: Q ratio should be 0.6, or that the hamstring should be 60% as strong as the quadriceps. ^32– 35^ The functional or mixed ratio of maximum eccentric knee flexion to maximum concentric knee extension (H: Q) has thus been established as being close to or over 1.0. ^36^ Studies also suggest that the forces generated by eccentric muscle movements are significantly greater than those generated by concentric muscle actions, especially at higher velocities, for preventing ACL and HSI. ^37, 38^ In our study, the findings suggested an FR of 51 %, 48%, and 42% for the Football, Cricket, and Tennis groups, respectively. Thus, it could be suggested that semi-professional players in the UAE have poor H/Q ratios and are prone to a higher risk of related injuries. Contributing factors warrant further studies to understand the variables that could have affected the isokinetic strengths of participants in the UAE, such as training techniques, practice hours, strength and conditioning behavior, lifestyle, food behaviors, etc. In support of our findings, a previously conducted study on athletes concluded a functional ratio of 0.7 during eccentric hamstring isokinetic testing against concentric quadriceps at 60°·s ^-1^. ^23^ Understandably, the ratio for Football could be higher since the player requires constant running with repetitive full knee extension. Comparatively, Tennis players would require a lesser eccentric activity of the Hamstring with a longer duration of knee flexion as per the demands of the sport. However, it should be noted that all three sports resemble the requirement for muscular balance between knee extensors and flexors. Thus H/Q ratio should be near 1 to reduce the risk of associated injuries, reflecting equal eccentric strength of the Hamstring against the concentric strength of the Quadriceps.

In addition to being necessary, having a higher H: Q ratio may also be beneficial in protecting the knee joint from injuries when significant forces are applied to it. According to a prior study, there could be a link between lower extremity injuries and low H: Q ratio. ^39– 41^ Hence, the present study shows that the risk of injury was more prominent in the Tennis group, followed by Cricket, and minimal in the Football group. The rationale is that a lower H/Q ratio would suggest that the Hamstring’s strength capacity may not be adequate to counter powerful Quadriceps contraction and the knee joint muscle integrity may be compromised, leading to a risk of injury. ^30^ In addition, other lower limb joints could be predisposed to injuries in a closed-chain kinematic function. A previous study investigated whether preseason isokinetic strength measures predicted future HSI among professional Football players. ^42^ It concluded that professional Football players with significantly lower isokinetic hamstring strength, lower hamstring-to-quadriceps strength ratio, and a previous injury of HSI were linked to an increased risk of recurrent HSI. ^42^ Additionally, a study evaluated the influence of Hamstrings and Quadriceps strength in male soccer players, and concluded that Hamstring strength deficit is the key factor for low H/Q ratios in male soccer players. ^43^

The peak isokinetic strengths for correlation between the groups were assessed as shown in Table 3. The correlation analyses revealed that the semi-professional athletes in all three sports groups demonstrated a weak correlation. This indicated that the functional H/Q ratio may not be related to the other. It was found that the H/Q ratio between the groups was statistically insignificant (p-value greater than 0.05, Table 3), suggesting that the physiological properties of one sport could not be transferred to another in terms of muscle strength. In other words, a good Tennis player without any history of injury could be at higher risk of HSI while playing Football since the demand for eccentric Hamstring strength would increase, as seen with a higher H/Q ratio in our study. It would be necessary to achieve an optimal H/Q ratio for semi-professional players to participate in multiple sports simultaneously, although training could be sports-specific. From the physical therapy perspective, the rehabilitation protocol for all three sports (Football, Cricket, and Tennis) would be different to attain a better FR. Further interpretation suggests that improving the H/Q ratio for Football may not benefit the player for Cricket or Tennis, as the muscle recruitment pattern may not be the same in all three sports groups. Hence, this strengthens the perspective that training benefits for one sport may not be transferred to another due to a weaker correlation in the H/Q ratio. A statistically non-significant correlation between the three sports groups affirms these findings, although the sports have similar demands of lower limb strength.

### Limitations

Only players from three comparable sports were involved in the study, which could be a potential limitation. Hence, studies considering players from other various forms of sports can be considered for the future scope of the study. Analysing senior and youth players in the same cohort could have affected the findings of the study. Multiple factors contribute to the strength of muscles, and these could be better explored through a longitudinal study.

## Conclusion

The isokinetic strength testing for H/Q ratio was found to be more in Football players, followed by Cricket and Tennis players for semi-professional male and female athletes. The study concluded that the functional H/Q ratio varies between three similar sports, as the demand on the target muscle would specifically vary. According to the findings of the study, it could be said that Cricket and Tennis players could have a higher risk for soft tissue injuries of the lower limb, attributed to a lower functional H/Q ratio compared to Football players. Necessary action and training strategies should be explored to achieve a higher H/Q ratio for isokinetic strength among these players. Also, a better isokinetic strength for Football skills may not impart similar physiological properties for Cricket and Tennis. Thus, semi-professional players who play multiple games should consider enhancing the FR for the Hamstring to Quadriceps, besides the strength and training specific to sports, to avoid the risk of injuries.

## Ethics approval and consent to participate

The study was conducted in line with the Declaration of Helsinki for human participants, and approved by the Institutional Review Board (IRB-COHS-STD-57-APRIL-2023), Gulf Medical University, Ajman, UAE, for ethical clearance.

## Consent to participate

A written informed consent was taken from all participants for their voluntary participation in the study before data collection, explaining the possible use of the data for research and publication without revealing their identity.

## Data Availability

The complete dataset has been given in the online repository. [Figshare]: [H/Q SEMIPROFESSIONAL UAE] [doi- 10.6084/m9.figshare.30156637] https://figshare.com/articles/dataset/H_Q_SEMIPROFESSIONAL_UAE/30156637. ^44^ Licence CC4.0 The project contains the following extended data: [Masterchat Thesis, Consent Form, IRB Approval] https://doi.org/10.6084/m9.figshare.30156637.v1. ^44^ Data are available under the terms of the CC BY 4.0 The manuscript is available as a preprint on the Research Square platform. ^45^
