# Supplementary material for: Isokinetic Strength and Functional Ratio for Quadriceps and Hamstrings among Semi-Professional Athletes in UAE playing Football, Cricket, and Tennis- Biomechanical Implications
Source: F1000Res. 2025 Oct 24;14:1163. [Version 1] doi: 10.12688/f1000research.170911.1 (PMC12800611; doi:10.12688/f1000research.170911.1)
Supplement: Peer review history

# Reviewer report: Reviewer response for version 1

**DOI:** 10.5256/f1000research.188430.r429956

**Published:** 2026-01-13

**Authors:** Asir John Samuel

**Affiliations:** 1 Yenepoya Physiotherapy College, Yenepoya(Deemed to be University), Mangaluru, Karnataka, India

The study aimed to compare the isokinetic strength of the Hamstrings against the Quadriceps for Football, Cricket, and Tennis among semi-professional athletes. Much needed research in present clinical scenario. Can be replicable in any clinical settings.

 Abstract:

 Methodology - Information related to sample design and correlational analysis to be added.

 Results - Demographic dimensions and correlational statistics to be added.

**Introduction:**

- Overall, the introduction is lengthy. Generally, the introduction should be 10% to a maximum of 20% of the total manuscript length.
  - Introduction should be drafted in 3 paragraphs. Please refer to the article for detailed description, (Reference 1)
    - *1 ^st^ Paragraph – Provide information about the general topic of the article in the light of the current literature which paves the way for the disclosure of the objective of the manuscript. (What is known?)*
    - *2 ^nd^ Paragraph – Focus on the specific subject matter, and the issue to be focused on should be dealt with, the problem should be brought forth, and fundamental references related to the topic should be discussed. (What is unknown?)*
    - *3 ^rd^ Paragraph - Finally, our recommendations for solution should be described, in other words our aim should be communicated. When these steps are followed in that order, the reader can track the problem, and its solution from his/her own perspective under the light of current literature. (What are you going to do?)*

Methodology:

 Study was conducted between? For how long? Time taken for collecting data from each sample?

 Sample size calculation is not appropriate. Use either sample size for correlation study (



 n=(Zα/2+Zβ)2/C2+3n equals open paren cap Z sub alpha / 2 end-sub plus cap Z sub beta close paren squared / cap C squared plus 3

 ��=(����/2+����)2 / C2 + 3) or comparison of three independent groups formula to be used.

 Statistical analysis, name of normalitytest - Shapiro-Wilk test to be added and confirmation message to state whether the data follow normal distribution or not to be clearly mentioned. "Pierson's" - to be corrected as, "Pearson's". Descriptions of various strength of correlation to be added.

 Results:

 Demographic dimensions such as, height, and weight in each group to be added in addion to BMI. Correlational graph in scatter plot with line of intersection to be added.

 Discussion:

 Avoid using numerical values in the discussion section.

 Conclusion:

 Conclusion section to be drafted in short two or three short sentences.

Is the work clearly and accurately presented and does it cite the current literature?

Yes

If applicable, is the statistical analysis and its interpretation appropriate?

Partly

Are all the source data underlying the results available to ensure full reproducibility?

Partly

Is the study design appropriate and is the work technically sound?

Yes

Are the conclusions drawn adequately supported by the results?

Yes

Are sufficient details of methods and analysis provided to allow replication by others?

Yes

Reviewer Expertise:

Sample size estimation and statistical analysis

I confirm that I have read this submission and believe that I have an appropriate level of expertise to confirm that it is of an acceptable scientific standard, however I have significant reservations, as outlined above.

## References

[1] : How to write an introduction section of a scientific article?. *Türk Üroloji Dergisi/Turkish Journal of Urology* .2014;39(1) : 10.5152/tud.2013.046 8-9 10.5152/tud.2013.046 PMC454856526328128

# Reviewer report: Reviewer response for version 1

**DOI:** 10.5256/f1000research.188430.r439535

**Published:** 2026-01-06

**Authors:** Jasmine Kaur Chawla

**Affiliations:** 1 Manav Rachna International Institute of Research and Studies, Faridabad, Haryana, India

This manuscript addresses an important and clinically relevant topic in sports biomechanics by comparing the functional hamstring-to-quadriceps (H/Q) ratio across three commonly played sports among semi-professional athletes in the UAE. The study is methodologically structured and contributes valuable normative data in a population that is underrepresented in the existing literature.

 The use of functional (eccentric hamstring to concentric quadriceps) ratios rather than conventional ratios strengthens the biomechanical relevance of the findings, particularly in relation to injury risk profiling. Overall, the manuscript is suitable for indexing minor corrections. 

 Observations: The conclusion that strength adaptations are not transferable across sports could be framed more cautiously. Please clarify that this inference is based on cross-sectional data and does not establish causality.

**Clarification of Sex-Based Analysis**

- Although male and female data are presented separately in Table 2, the manuscript would benefit from a clearer rationale for the gender-based comparisons.
  - Please clarify whether the study was powered to detect sex differences.
  - Consider adding a brief interpretation of sex-specific findings in the Discussion

**Terminology Consistency**

 Ensure consistent use of terms such as “functional ratio (FR)” and “H/Q ratio” throughout the manuscript to avoid confusion.

Is the work clearly and accurately presented and does it cite the current literature?

Yes

If applicable, is the statistical analysis and its interpretation appropriate?

Yes

Are all the source data underlying the results available to ensure full reproducibility?

Yes

Is the study design appropriate and is the work technically sound?

Yes

Are the conclusions drawn adequately supported by the results?

Yes

Are sufficient details of methods and analysis provided to allow replication by others?

Yes

Reviewer Expertise:

biomechanics, sport injuries, prediction model specialist, women health, exercise physiology, sports physioitherapy

I confirm that I have read this submission and believe that I have an appropriate level of expertise to confirm that it is of an acceptable scientific standard.

# Reviewer report: Reviewer response for version 1

**DOI:** 10.5256/f1000research.188430.r429954

**Published:** 2025-12-28

**Authors:** Miglena Tsvetkova-Gaberska, Maria Ganeva

**Affiliations:** 1 South-West University, Blagoevgrad, Bulgaria; 2 Anatomy and Physiology, South-west University, Blagoevgrad, Blagoevgrad, Bulgaria

The study is interesting and well presented. However, the methodology lacks information on how the ratio between H/Q was calculated, what the normative values set in the isokinetic dynamometer software are, and what the criteria for unilateral asymmetry are. How do competitors from individual disciplines compare to the norms? It would be beneficial to examine both legs, as the supporting leg plays a crucial role in the accuracy of sports-related tasks, and to calculate bilateral asymmetry as a predictor of trauma and injury. Another important point regarding the results is why the data for peak torque is not presented in newton metres (Nm). In terms of the physiology of the quadriceps and hamstrings, it is well known that the knee extensors are stronger than the knee flexors, with a ratio of 60:40. This is not a novel finding. It is also evident that women have weaker strength and joint stability than men. The specificity of each sport determines the type of load, and a lack of proper training, especially with regard to hamstring exercises, can be assumed based on the research. To clarify the design and get into the specifics, it would be useful to compare the studied groups only by gender (men with men; women with women). Overall, the manuscript presents very general information and would benefit from going into more detail and comparing the data with normative values to be more useful for athletes and to make recommendations. We would also recommend studying speeds of 180 and 300 to gain a more complete picture of isokinetic dynamic loads and to better reflect the dynamics of specific sports. Of the sources cited in the literature, over 30 are more than 5 years old, so it would be beneficial to refer to more recent research, of which there is plenty in global databases.

Is the work clearly and accurately presented and does it cite the current literature?

Partly

If applicable, is the statistical analysis and its interpretation appropriate?

I cannot comment. A qualified statistician is required.

Are all the source data underlying the results available to ensure full reproducibility?

Partly

Is the study design appropriate and is the work technically sound?

Partly

Are the conclusions drawn adequately supported by the results?

Partly

Are sufficient details of methods and analysis provided to allow replication by others?

Yes

Reviewer Expertise:

Kinesiology, Biomechanics, Kinesitherapy, Physiotherapy, Sport Injuries, Isokinetic Dynamometry

We confirm that we have read this submission and believe that we have an appropriate level of expertise to confirm that it is of an acceptable scientific standard, however we have significant reservations, as outlined above.
